# Eye-related emergency visits during the early phase of the coronavirus disease pandemic in a reference hospital in Sao Paulo, Brazil

**DOI:** 10.5935/0004-2749.20230041

**Published:** 2023

**Authors:** Evandro Schapira, Rodrigo Antonio Brant Fernandes, Arthur Gustavo Fernandes

**Affiliations:** 1 Ophthal Hospital Especializado Ltda, São Paulo, SP, Brazil.; 2 Departamento de Oftalmologia e Ciências Visuais, Escola Paulista de Medicina, Universidade Federal de São Paulo, São Paulo, SP, Brazil.

**Keywords:** COVID-19, Emergencies, Social Isolation, Ophthalmology, Quarantine, COVID-19, Emergências, Isolamento Social, Oftalmologia, Quarentena

## Abstract

**Purpose:**

To evaluate the profile of emergency eye-related visits at a reference eye hospital in Sao Paulo during the first months of the quarantine due to the coronavirus disease (COVID-19) pandemic and to compare it with that in the same period of the previous year.

**Methods:**

Data were obtained from the emergency department of Ophthal Hospital Especializado, Sao Paulo, Brazil. All the cases registered between March 23 and May 19, 2020, were included in the study as Group 2020. The cases registered between March 23 and May 19, 2019, were included in the study as Group 2019. Frequency tables were used for the descriptive analyses. The chi-square and Fisher exact tests were applied to compare categorical variables between the groups.

**Results:**

We observe a decrease of 46.15% in the number of cases during the COVID-19 pandemic in 2020 compared with the same period in 2019. We observed a significant increase in the incidence rates of the following pathologies in 2020 compared with 2019: eyelid disorders (12.3%), corneal disorders (97.1%), retinal pathologies (173.1%), refraction (62.9%), glaucoma (acute and chronic; 43.9%), scleral alterations (68.8%), trauma (39.3%), herpes (54.7%), and cataracts (549.9%). On the other hand, the incidence rates of the following disorders decreased: conjunctivitis (-33.4%), disorders of lacrimal system (-81.0%), iridocyclitis (-39.9%), and postoperative visits (-80.1%).

**Conclusion:**

During the early phase of the COVID-19 pandemic, we observed a drastic decrease in the number of patients who visited the emergency eye service. The main reasons for visiting were also changed, with higher frequencies of high-severity cases such as retina disturbances, cornea disturbances, glaucoma, and trauma and lower frequencies of transmittable conditions such as conjunctivitis.

## Abstract (pt)

**Objetivos:**

Avaliar o perfil das consultas de emergência oftalmológicas durante os primeiros meses de quarentena por pandemia de covid-19 em um hospital oftalmológico de referência em São Paulo e compará-lo com o mesmo período do ano anterior.

**Métodos:**

Os dados foram extraídos do serviço de emergência do Hospital Ophthal Especializado, São Paulo, Brasil. Todos os casos registrados entre 23 de março e 19 de maio de 2020 foram incluídos no estudo como Grupo 2020. Os casos registrados entre 23 de março e 19 de maio de 2019 foram incluídos no estudo como Grupo de 2019. Tabelas de frequência foram utilizadas para análises descritivas. Os testes Qui-quadrado e Exato de Fisher foram aplicados para comparar variáveis categóricas entre os grupos.

**Resultados:**

Observamos uma diminuição de 46,15% no número de casos durante a pandemia Covid-19 em 2020 quando comparado ao mesmo período sem pandemia em 2019. Foi observado um aumento significativo nos achados de pálpebra (+12,3%), córnea (+97,1%), retina (+173,1%), refração (+62,9%), glaucoma (+43,9%), esclera (+68,8%), trauma (+39,3%), herpes (+54,7%) e catarata (+549,9%) em 2020 quando comparado para 2019. Por outro lado, houve redução nos casos de conjuntivite (-33,4%), transtornos do aparelho lacrimal (-81,0%), iridociclite (-39,9%) e consultas pós-operatórias (-80,1%).

**Conclusão:**

durante a fase inicial da pandemia Covid-19, foi observada uma redução drástica no número de pacientes que procuram o serviço de emergência ocular. Houve também uma mudança nos principais motivos para visitas com frequências mais altas de casos de alta gravidade como distúrbios da retina, distúrbios da córnea, glaucoma e trauma, e frequências mais baixas de condições transmissíveis como conjuntivite.

## INTRODUCTION

Eye-related emergency services are uncommon in most places, although their importance is undeniable, as most general physicians are not prepared to provide ocular treatment and management^(1)^.

In December 2019, the first cases of respiratory failure of unknown cause were reported in Wuhan, Hubei, China. In February 2020, the World Health Organization (WHO) named this new entity COVID-19. Caused by a type of coronavirus, the main clinical features of the disease include respiratory symptoms and fever, but fatigue, myalgia, and diarrhea, among others, can also be reported^(2,3)^.

In Brazil, the first case of COVID-19 was confirmed in February 26, 2020, and the pandemic was declared on March 11. On June 19, the number of COVID-19 cases in the country reached 1 million^(4)^. As part of the strategies to control the disease spread, social distancing was strongly recommended by the WHO, which impacted different sections of society in all aspects.

As a result of the social distancing policies, the number of people who attended hospitals and health centers, especially for non-emergency situations, decreased^(5)^. The American Association of Ophthalmology has even recommended that ophthalmologists avoid patient examinations and surgical interventions except for emergency cases to maintain social distance^(6)^.

The purpose of this study was to evaluate the profile of emergency eye-related visits at a reference eye hospital in Sao Paulo during the first months of the quarantine due to the COVID-19 pandemic and to compare it with that in the same period of the previous year.

## METHODS

Data were obtained from the emergency department of Ophthal Hospital Especializado, Sao Paulo, Brazil. All the cases registered between March 23 and May 19, 2020, were included in the study as Group 2020. The cases registered between March 23 and May 19, 2019, were included in the study as Group 2019. All the cases were classified in accordance with the *International Classification of Diseases, Tenth Revision,* codes and grouped into broader groups for analysis.

Statistical analyses were performed using the Stata/SE Statistical Software, Release 14.0, 2015 (Stata Corp, College Station, Texas, USA). Frequency tables were used for the descriptive analyses. The chi-square and Fisher exact tests were applied to compare categorical variables between the groups. For all the tests, p values <0.05 were considered statistically significant.

The study protocol was approved by the Hospital Paulista Reviewer Boards and conducted in accordance with the tenets of the Declaration of Helsinki.

## RESULTS

A total of 6,071 visits were included in the study, 3,946 in 2019 and 2,125 in 2020. These number represents a decrease of 46.15% during the COVID-19 pandemic in 2020. Table 1 shows the profiles of the individuals who visited the service in the two periods analyzed.

**Table 1:** Participants’ demographics

	2019	2020	
	n (%)	n (%)	p Value
**Sex**			
Male	1941 (49.19)	1130 (53.18)	0.003
Female	2005 (50.81)	995 (46.82)	
**Age category**			
0-19 years	511 (12.95)	196 (9.22)	<0.001
20-59 years	2891 (73.26)	1573 (74.02)	
≥60 years	544 (13.79)	356 (16.75)	
**TOTAL**	**3946**	**2125**	

The chi-square test shows a higher frequency of men, a lower frequency of patients aged 0 to 19 years, and a higher frequency of patients aged ≥60 years in 2020 than in 2019. Table 2 shows the reasons for the visits in 2019 and 2020.

**Table 2:** Reasons for visiting the eye emergency service

	2019	2020	Change
	n (%)	n (%)	(%)
**Conjunctival disturbances**	1488 (37.71)	534 (25.13)	-33.36
**Eyelid disturbances**	635 (16.09)	384 (18.07)	+12.29
**Corneal disturbances**	523 (13.25)	555 (26.12)	+97.06
**Retina disturbances**	85 (2.15)	125 (5.88)	+173.08
**Refraction**	57 (1.44)	50 (2.35)	+62.89
**Glaucoma**	40 (1.01)	31 (1.46)	+43.91
**Disorders of the lacrimal system**	39 (0.99)	4 (0.19)	-80.95
**Iridocyclitis**	34 (0.86)	11 (0.52)	-39.92
**Scleral disturbances**	33 (0.84)	30 (1.41)	+68.81
**Trauma**	28 (0.71)	21 (0.99)	+39.27
**Postoperative visits**	28 (0.71)	3 (0.14)	-80.10
**Herpes**	18 (0.46)	15 (0.71)	+54.75
**Cataracts**	8 (0.20)	28 (1.32)	+549.93
**Orbit disturbances**	4 (0.10)	4 (0.19)	+85.69
**Other causes**	132 (3.35)	155 (7.29)	+118.05
**Unspecific**	794 (20.12)	175 (8.24)	-59.07
**TOTAL**	**3946 (100.00)**	**2125 (100.00)**	

The incidence rates of the following pathologies were significantly increased in 2020 as compared with 2019: eyelid, corneal, and retinal diseases; refraction; glaucoma; sclera; trauma; herpes; and cataracts. On the other hand, the incidence rates of conjunctiva, disorders of the lacrimal system, iridocyclitis, and postoperative visits decreased.

## DISCUSSION

Our results show a drastic decrease in the number of patients who visited the emergency eye service during the COVID-19 quarantine (46.15%), despite the service availability that remained opened 24 hours per day. Previous reports on ophthalmology outpatients from reference hospitals decreased up to 72.5%^(7-9)^. Other specialties also showed decreases in the emergency service attendance^(10,11)^. A previous study conducted in the UK during the same period showed a decrease of 53.1%, with similar trends on the reasons for attendance in the service^(12)^.

The main reasons for the reduction in the number of visits in our study were the lower number of cases classified as unspecific (59%) and conjunctivitis (33%). Unspecific reasons included emergency service visits without any real clinical motivation to justify them. Most of the time, the individuals classified under this category visit the emergency service to obtain a medical certificate to justify their absence from work. In the pandemic scenario, with the imposition of social distancing, several people were already working on a home-office scheme, which contributed to the decrease in unspecific visits. On the other hand, the decrease in the incidence rate of conjunctivitis was a positive effect of the quarantine, as people tended to improve their hygiene habits and avoid contact with each other, reducing the conjunctive spread^(13)^.

In response, significant increases in the incidence rates of retinal disturbances, corneal disturbances, glaucoma, and trauma were observed, indicating that cases that in fact demand emergency response remained present during the pandemic, showing a higher frequency as the total number of visits decreased. Among the corneal disorders, we highlight keratitis caused by alcohol gel in children (aged <8 years)^(14)^. The disease was rare in our service; for example, in the studied period in 2019, no case was observed, whereas in the same period in 2020, four cases occurred, which can be explained by the parents’ carelessness in allowing the handling of gel alcohol by children, a new routine during the pandemic.

During the pandemic, especially in the first months, elective surgeries (i.e., cataract and refractive, among others) were postponed mainly because patients opted for the postponement. Therefore, we observed a lower demand for emergency service visits by postoperative patients^(4)^.

With both surgeries and elective appointments canceled during the first months of the pandemic, patients who would normally seek for regular consultancy with an ophthalmologist ended up visiting the emergency service, even for non-emergency reasons, owing to the unavailability of other services. Thus, increases in the incidence rates of cataract and refraction were observed in our analysis. For other disorders such as those related to the lacrimal system (e.g., dry eyes and epiphora), being usually chronic pathologies and not sight threatening, patients could wait for an elective consultation, which could explain the decrease of 80% in the consultancy rate.

Clearly, this difficult period induced feelings of anguish, stress, lack of perspective, fear of the future, and impotence, causing pathologies related in some way to the emotional state explaining the increases in the incidence rates of eyelid disorders (hordeolum and chalazion), corneal disorders (herpetic keratitis), and facial herpes zoster^(15,16)^.

In conclusion, during the early phase of the COVID-19 pandemic, we observed a drastic decrease in the number of patients visiting the emergency eye service. The main reasons for visiting were also changed, with higher incidence rates of high-severity cases such as retinal disturbances, corneal disturbances, glaucoma, and trauma, and lower incidence rates of transmittable conditions such as conjunctivitis.
